# Crystal structure, Hirshfeld surface analysis and density functional theory study of benzyl 2-oxo-1-(prop-2-yn-1-yl)-1,2-di­hydro­quinoline-4-carboxyl­ate

**DOI:** 10.1107/S2056989021007416

**Published:** 2021-07-23

**Authors:** Younos Bouzian, Karim Chkirate, Joel T. Mague, Fares Hezam Al-Ostoot, Noureddine Hammou Ahabchane, El Mokhtar Essassi

**Affiliations:** a Laboratory of Heterocyclic Organic Chemistry URAC 21, Pharmacochemistry Competence Center, Av. Ibn Battouta, BP 1014, Faculty of Sciences, Mohammed V University, Rabat, Morocco; b Department of Chemistry, Tulane University, New Orleans, LA 70118, USA; c Department of Biochemistry, Faculty of Education & Science, Al-Baydha University, Yemen

**Keywords:** crystal structure, alkyne, di­hydro­quinoline, hydrogen bond, Hirshfeld surface analysis

## Abstract

The title mol­ecule, C_20_H_15_NO_3_, adopts a *Z*-shaped conformation with the carboxyl group nearly coplanar with the di­hydro­quinoline unit. In the crystal, corrugated layers are formed by C—H⋯O hydrogen bonds and are stacked by C—H⋯π(ring) inter­actions. Hirshfeld surface analysis indicates that the most important contributions to the crystal packing are from H⋯H (43.3%), H⋯C/C⋯H (26.6%) and H⋯O/O⋯H (16.3%) inter­actions. The optimized structure calculated using density functional theory at the B3LYP/ 6–311 G(d,p) level is compared with the experimentally determined structure in the solid state. The calculated HOMO–LUMO energy gap is 4.0319 eV.

## Abstract 2

The mol­ecule adopts a *Z*-shaped conformation with the carboxyl group nearly coplanar with the di­hydro­quinoline unit. In the crystal, two sets of C—H⋯O hydrogen bonds form chains along the *b*-axis direction, which are connected into corrugated layers parallel to (103) by additional C—H⋯O hydrogen bonds. The layers are connected by C—H⋯π(ring) inter­actions.

## Chemical context   

Nitro­gen-based structures have attracted increased attention in recent years because of their inter­esting properties in structural and inorganic chemistry (Chkirate *et al.*, 2019 ▸, 2020*a* ▸,*b* ▸, 2021 ▸). The family of quinolines, particularly those containing the 2-oxo­quinoline moiety, is important in medicinal chemistry because of their wide range of pharmacological applications including as potential anti­cancer agents (Fang *et al.*, 2021 ▸), anti-proliferative agents (Banu *et al.*, 2017 ▸) and as potent modulators of ABCB1-related drug resistance of mouse T-lymphoma cells (Filali Baba *et al.*, 2020 ▸). In particular, 2-oxo­quinoline-4-carboxyl­ate derivatives are active anti­oxidants (Filali Baba *et al.*, 2019 ▸). Given the wide range of therapeutic applications for such compounds, and in a continuation of the work already carried out on the synthesis of compounds resulting from quinolin-2-one (Bouzian *et al.*, 2020 ▸), a similar approach gave the title compound, benzyl 2-oxo-1-(prop-2-yn-1-yl)-1,2-di­hydro­quinoline-4-carboxyl­ate, (I). Besides the synthesis, we also report the mol­ecular and crystalline structures along with a Hirshfeld surface analysis and a density functional theory computational calculation carried out at the B3LYP/6– 311 G(d,p) level. 
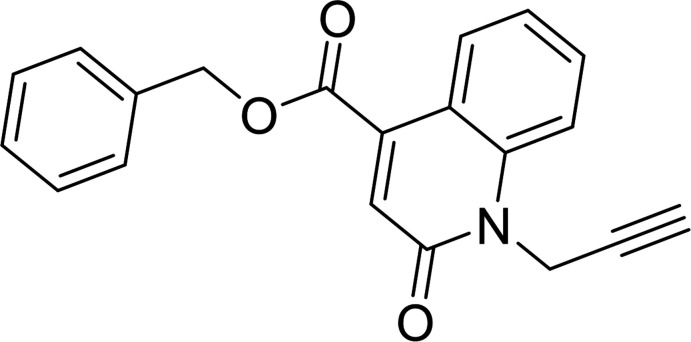


## Structural commentary   

**Figure 1:**
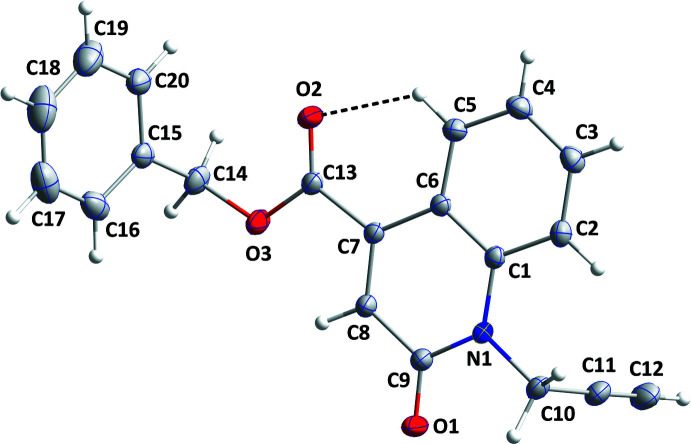
The title mol­ecule with labeling scheme and 50% probability ellipsoids. The intra­molecular hydrogen bond is depicted by a dashed line.

The mol­ecule adopts a Z-shaped conformation with the propynyl and benzyl substituents projecting from opposite sides of the mean plane of the di­hydro­quinoline moiety. This moiety is planar to within 0.0340 (6) Å (r.m.s. deviation = 0.0164) with N1 and C9 being, respectively, 0.0340 (6) and −0.0279 (7) Å from the mean plane, resulting in a slight twist at this location. The carboxyl group is nearly coplanar with the di­hydro­quinoline as seen from the 1.04 (5)° dihedral angle between the plane defined by C7/C13/O2/O3 and that of the di­hydro­quinoline (C1–C9/N1/O1). This is likely due, in part, to the intra­molecular C5—H5⋯O2 inter­action (Table 1 ▸ and Fig. 1 ▸). The propynyl substituent is rotated out of the mean plane of the di­hydro­quinoline moiety by 80.88 (3)°. The plane of the C15–C20 ring is inclined to that of the di­hydro­quinoline by 68.47 (2)°.

## Supra­molecular features   

**Figure 2:**
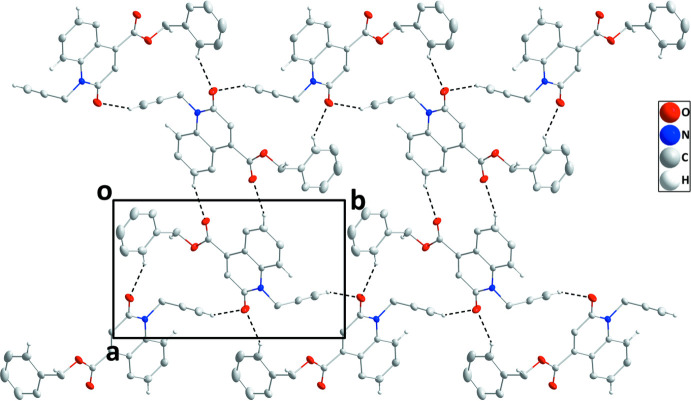
A portion of one layer viewed along the *c* axis with C—H⋯O hydrogen bonds depicted by dashed lines.

**Figure 3:**
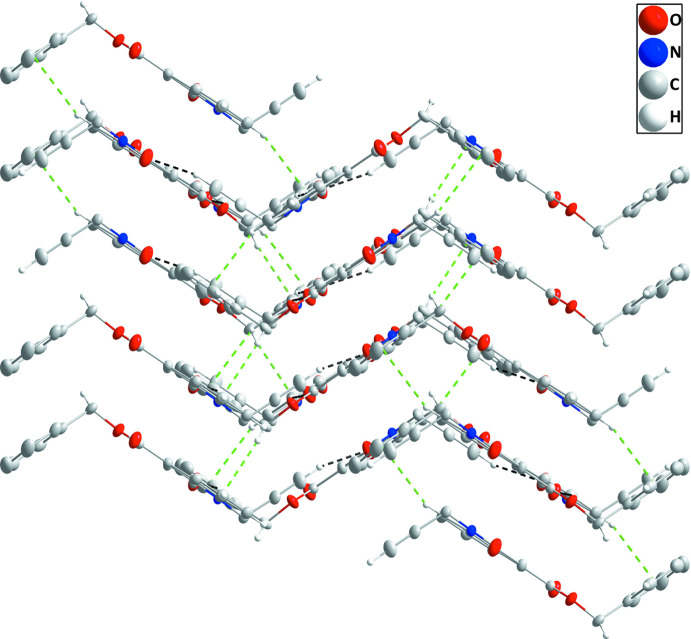
Packing viewed parallel to (103) with the *b* axis horizontal and running from left to right. C—H⋯O hydrogen bonds and C—H⋯π(ring) inter­actions are depicted, respectively, by black and green dashed lines.

In the crystal, C12—H12⋯O1 and C16—H16⋯O1 hydrogen bonds (Table 1 ▸) link the mol­ecules into zigzag chains extending along the *b*-axis direction, which are connected by inversion-related pairs of C4—H4⋯O2 hydrogen bonds (Table 1 ▸) into corrugated layers parallel to the (103) plane (Fig. 2 ▸). The layers are stacked along the normal to (103) with C2—H2⋯*Cg*3 and C14—H14*A*⋯*Cg*2 inter­actions (Table 1 ▸ and Fig. 3 ▸).

## Hirshfeld surface analysis   

**Figure 4:**
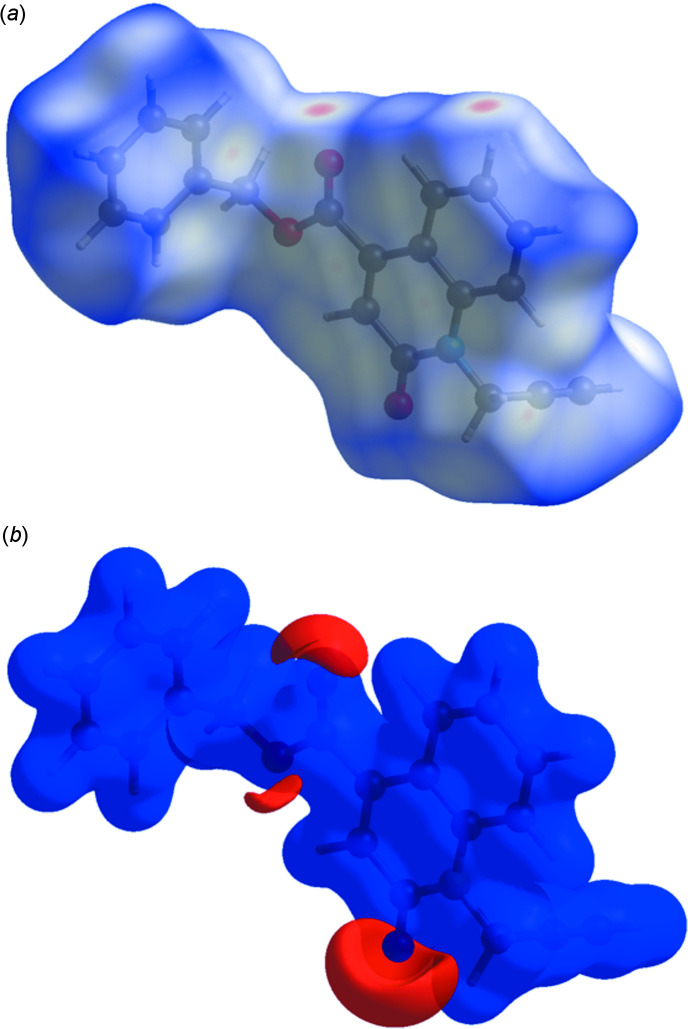
(*a*) View of the three-dimensional Hirshfeld surface of the title compound, plotted over *d*_norm_ in the range of −0.3677 to 1.3896 a.u. (*b*) View of the three-dimensional Hirshfeld surface of the title compound plotted over electrostatic potential energy in the range −0.0500 to 0.0500 a.u. using the STO-3 G basis set at the Hartree–Fock level of theory.

The *CrystalExplorer* program (Turner *et al.*, 2017 ▸) was used to investigate and visualize further the inter­molecular inter­actions of (I). The Hirshfeld surface plotted over *d*_norm_ in the range −0.3677 to 1.3896 a.u. is shown in Fig. 4 ▸ *a*. The electrostatic potential using the STO-3G basis set at the Hartree–Fock level of theory and mapped on the Hirshfeld surface over the range of ±0.05 a.u. clearly shows the positions of close inter­molecular contacts in the compound (Fig. 4 ▸ *b*). The positive electrostatic potential (blue region) over the surface indicates hydrogen-donor potential, whereas the hydrogen-bond acceptors are represented by negative electrostatic potential (red region).

**Figure 5:**
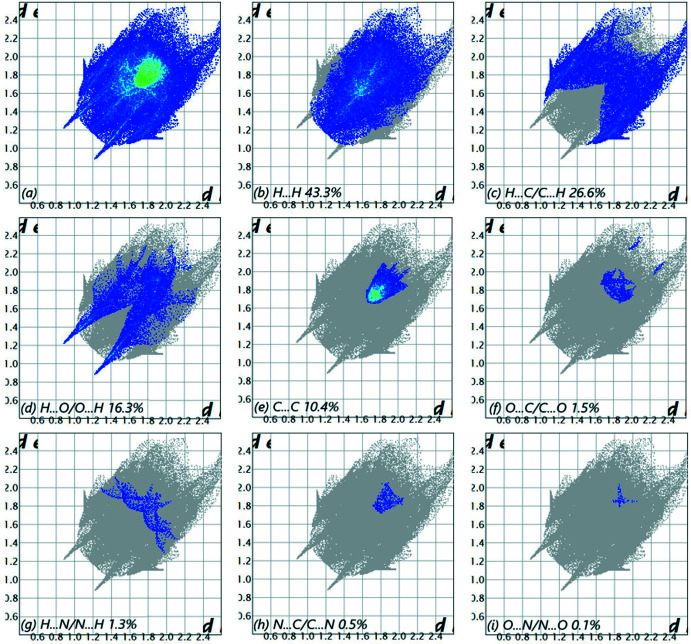
The full two-dimensional fingerprint plots for the title compound, showing (*a*) all inter­actions, and delineated into (*b*) H⋯H, (*c*) H⋯C/C⋯H, (*d*) H⋯O/O⋯H, (*e*) C⋯C, (*f*) O⋯C/C⋯O, (*g*) H⋯N/N⋯H, (*h*) N⋯C/C⋯N and (i) N⋯O/O⋯N inter­actions. *d_i_* and *d_e_* values are the closest inter­nal and external distances (in Å) from given points on the Hirshfeld surface.

The overall two-dimensional fingerprint plot (McKinnon *et al.*, 2007 ▸) is shown in Fig. 5 ▸ *a*, while those delineated into H⋯H, H⋯C/C⋯H, H⋯O/O⋯H, C⋯C, O⋯C/C⋯O, H⋯N/N⋯H, N⋯C/C⋯N and N⋯O/O⋯N contacts are illustrated in Fig. 5 ▸ *b*–*i*, respectively, together with their relative contributions to the Hirshfeld surface (HS). The most important inter­action is H⋯H, contributing 43.3% to the overall crystal packing, which is reflected in Fig. 5 ▸ *b* as widely scattered points of high density due to the large hydrogen content of the mol­ecule, with its tip at *d*_e_ = *d*_i_ = 1.19 Å. In the presence of C—H inter­actions, the pair of characteristic wings in the fingerprint plot delineated into H⋯C/C⋯H contacts (26.6% contribution to the HS, Fig. 5 ▸ *c*) has tips at *d*_e_ + *d*_i_ = 3.07 Å. The pair of scattered points of spikes in the fingerprint plot delineated into H⋯O/O⋯H contacts (Fig. 5 ▸ *d*, 16.3%) have tips at *d*_e_ + *d*_i_ = 2.08 Å. The C⋯C contacts (Fig. 5 ▸ *e*, 10.4%) have tips at *d*_e_ + *d*_i_ = 3.34 Å. The O⋯C/C⋯O contacts, Fig. 5 ▸ *f*, contribute 1.5% to the HS and appear as a pair of scattered points of spikes with tips at *d*_e_ + *d*_i_ = 3.55 Å. The H⋯N/N⋯H contacts (Fig. 5 ▸ *g*, 1.3%) have tips at *d*_e_ + *d*_i_ = 3.28 Å. Finally, the C⋯N/N⋯C and O⋯N/N⋯ O contacts, Fig. 5 ▸ *h*–*i*, contribute only 0.5% and 0.1% respectively to the HS and have a low-density distribution of points.

## Density Functional Theory calculations   

**Figure 6:**
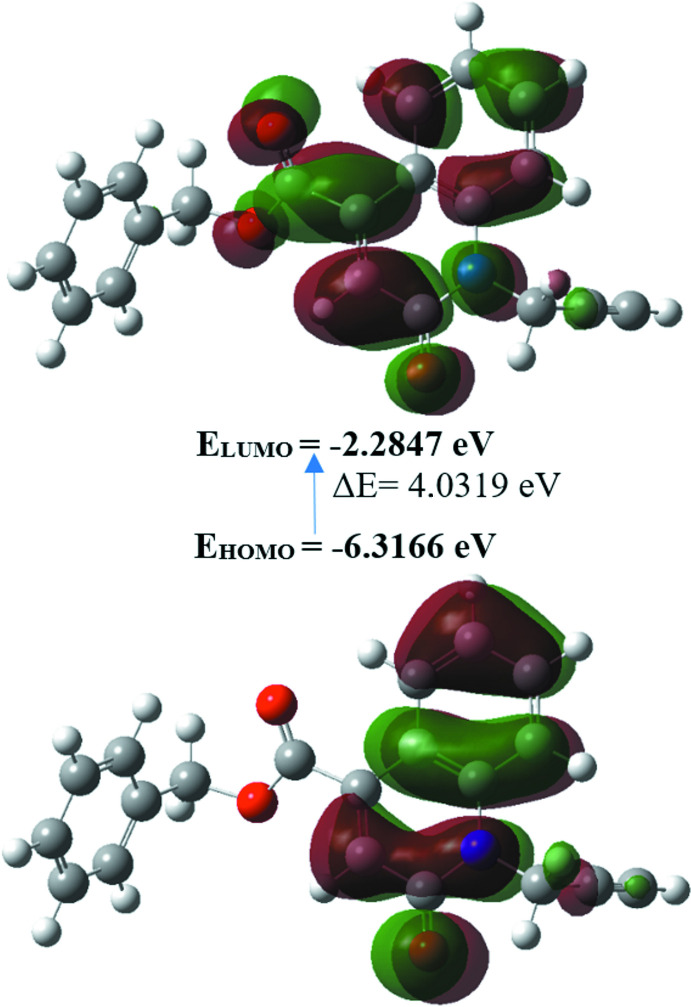
The energy band gap of benzyl 2-oxo-1-(prop-2-yn-1-yl)-1,2-di­hydro­quinoline-4-carboxyl­ate.

The structure in the gas phase of the title compound was optimized by means of density functional theory. The density functional theory calculation was performed by the hybrid B3LYP method and the 6–311 G(d,p) basis-set, which is based on Becke’s model (Becke, 1993 ▸) and considers a mixture of the exact (Hartree–Fock) and density functional theory exchange utilizing the B3 functional, together with the LYP correlation functional (Lee *et al.*, 1988 ▸). After obtaining the converged geometry, the harmonic vibrational frequencies were calculated at the same theoretical level to confirm that the number of imaginary frequencies is zero for the stationary point. Both the geometry optimization and harmonic vibrational frequency analysis of the title compound were performed with the *Gaussian 09* program (Frisch *et al.*, 2009 ▸). Theoretical and experimental results related to bond lengths and angles are in good agreement, and are summarized in Table 2 ▸. Calculated numerical values for the title compound including electronegativity (*χ*), hardness (*η*), ionization potential (*I*), dipole moment (*μ*), electron affinity (*A*), electrophilicity (*ω*) and softness (*σ*) are collated in Table 3 ▸. The electron transition from the highest occupied mol­ecular orbital (HOMO) to the lowest unoccupied mol­ecular orbital (LUMO) energy level is shown in Fig. 6 ▸. The HOMO and LUMO are localized in the plane extending over the whole benzyl 2-oxo-1-(prop-2-yn-1-yl)-1,2-di­hydro­quinoline-4-carb­oxyl­ate system. The energy band gap (*ΔE* = *E*_LUMO_ − *E*_HOMO_) of the mol­ecule is 4.0319 eV, and the frontier mol­ecular orbital energies, *E*_HOMO_ and *E*_LUMO_, are −6.3166 and −2.2847 eV, respectively.

## Database survey   

A search of the Cambridge Structural Database (CSD version 5.42, updated May 2021; Groom *et al.*, 2016 ▸) with the 2-oxo-1-(prop-2-yn-1-yl)-1,2-di­hydro­quinoline-4-carboxyl­ate fragment yielded multiple matches. Of these, two had an alkyl substituent on O3 comparable to (I). The first compound (refcode OKIGAT; Hayani *et al.*, 2021 ▸) carries an ethyl group on O3, while the second one (refcode OKIGOH; Hayani *et al.*, 2021 ▸) carries a cyclo­hexyl group. The ethyl carboxyl­ate in OKIGAT forms a dihedral angle of −8.3 (7)° with the di­hydro­quinoline unit. In OKIGOH, the dihedral angle between the mean planes of the cyclo­hexyl carboxyl­ate and di­hydro­quinoline rings is 37.3 (8)°. As previously mentioned, the carboxyl group in (I) is nearly coplanar with the di­hydro­quinoline [dihedral angle of 1.04 (5)°], which is approximately the same as in OKIGAT, but less tilted than in OKIGOH.

## Synthesis and crystallization   

A mixture of 2-oxo-1-(prop-2-yn-1-yl)-1,2-di­hydro­quinoline-4-carb­oxy­lic acid (0.7 g, 3 mmol), K_2_CO_3_ (0.51 g, 3.6 mmol), benzyl chloride (0.76 ml, 6 mmol) and tetra *n*-butyl­ammonium bromide as a catalyst in DMF (30 mL) was stirred at room temperature for 48 h. After removal of the salts by filtration, the solvent was evaporated under reduced pressure and the residue obtained was dissolved in di­chloro­methane. The organic phase was dried over Na_2_SO_4_ and concentrated under vacuum. The crude product obtained was purified by chromatography on a column of silica gel (eluent: hexa­ne/ ethyl acetate: 9/1). ^1^H NMR (300 MHz, DMSO-*d*_6_) δ ppm: 3.08 (*t*, 1H, CH≡); 4.37 (*d*, 2H, CH_2_—N); 5.12 (*s*, 2H, CH_2_—O); 7.08–8.74 (*m*, 10H, CH_arom_); ^13^C NMR (75 MHz, DMSO-*d*_6_) δ ppm: 34.3 (CH_3_—N); 66.2 (CH_2_—O); 72.1 (–C≡); 73.2 (CH≡); 115.6-148.7 (CH_arom_ and C_quat arom_); 162. 5 (C=O_quinol_); 168.2 (C=O_carbox­yl_). MS (ESI): *m*/*z* = 318 (*M* + H)^+^.

## Refinement   

Crystal data, data collection and structure refinement details are summarized in Table 4 ▸. H atoms attached to carbon were placed in calculated positions (C—H = 0.95–1.00 Å), and were included as riding contributions with isotropic displacement parameters 1.2 or 1.5 times those of the attached atoms. Two reflections affected by the beamstop were omitted from the final refinement.

## Supplementary Material

Crystal structure: contains datablock(s) global, I. DOI: 10.1107/S2056989021007416/tx2040sup1.cif

Structure factors: contains datablock(s) I. DOI: 10.1107/S2056989021007416/tx2040Isup3.hkl

Supporting information file. DOI: 10.1107/S2056989021007416/tx2040Isup3.cml

CCDC reference: 2097267

Additional supporting information:  crystallographic information; 3D view; checkCIF report

## Figures and Tables

**Table 1:** Hydrogen-bond geometry (Å, °) *Cg*2 and *Cg*3 are the centroids of the C1–C6 and C15–C20 benzene rings, respectively.

*D*—H⋯*A*	*D*—H	H⋯*A*	*D*⋯*A*	*D*—H⋯*A*
C2—H2⋯*Cg*3^i^	0.95	2.94	3.8206 (10)	154
C4—H4⋯O2^ii^	0.95	2.57	3.4846 (11)	162
C5—H5⋯O2	0.95	2.23	2.8917 (11)	126
C12—H12⋯O1^iii^	0.95	2.25	3.1463 (14)	157
C14—H14*A*⋯*Cg*2^iv^	0.99	2.65	3.4652 (9)	140
C16—H16⋯O1^v^	0.95	2.50	3.3443 (12)	148

Symmetry codes: (i) -x+1, -y+1, -z+1; (ii) -x, -y+1, -z+1; (iii) -x+{\script{3\over 2}}, y+{\script{1\over 2}}, -z+{\script{1\over 2}}; (iv) -x+{\script{1\over 2}}, y-{\script{1\over 2}}, -z+{\script{1\over 2}}; (v) -x+{\script{3\over 2}}, y-{\script{1\over 2}}, -z+{\script{1\over 2}}.

**Table 2:** Comparison (X-ray and DFT) of selected bond lengths and angles (Å, °)

	X-ray	B3LYP/6–311G(d,p)
O1—C9	1.2355 (10)	1.223
O3—C13	1.3375 (10)	1.3447
N1—C9	1.3788 (10)	1.4042
N1—C10	1.4730 (10)	1.4725
O2—C13	1.2058 (10)	1.2092
O3—C14	1.4588 (10)	1.4611
N1—C1	1.3999 (10)	1.3953
		
C13—O3—C14	116.87 (7)	117.1258
C9—N1—C10	115.85 (6)	115.6313
N1—C1—C2	119.87 (7)	120.5532
O1—C9—N1	121.42 (7)	121.7499
N1—C9—C8	116.04 (7)	115.2168
O2—C13—C7	125.74 (7)	125.0357
O3—C14—C15	112.63 (7)	111.678
C9—N1—C1	123.16 (6)	123.4431
C1—N1—C10	120.93 (6)	120.911
N1—C1—C6	120.08 (6)	120.1155
O1—C9—C8	122.54 (7)	123.0317
C11—C10—N1	111.46 (7)	113.9875
O2—C13—O3	123.21 (7)	123.6586
O3—C13—C7	111.05 (6)	111.3015

**Table 3:** Calculated energies

Mol­ecular energy	Compound (I)
Total energy *TE* (eV)	−28621.0571
*E*_HOMO_ (eV)	−6.3166
*E*_LUMO_ (eV)	−2.2847
Gap, *ΔE* (eV)	4.0319
Dipole moment, *μ* (Debye)	1.9469
Ionization potential, *I* (eV)	6.3166
Electron affinity, *A*	2.2847
Electronegativity, *χ*	4.3007
Hardness, *η*	2.0160
Electrophilicity index, *ω*	4.5873
Softness, *σ*	0.4960
Fraction of electron transferred, *ΔN*	0.6695

**Table 4:** Experimental details

Crystal data	
Chemical formula	C_20_H_15_NO_3_
*M*_r_	317.33
Crystal system, space group	Monoclinic, *P*2_1_/*n*
Temperature (K)	150
*a*, *b*, *c* (Å)	8.2284 (3), 13.7693 (4), 13.9230 (4)
β (°)	96.155 (1)
*V* (Å^3^)	1568.37 (9)
*Z*	4
Radiation type	Mo *K*α
μ (mm^−1^)	0.09
Crystal size (mm)	0.44 × 0.35 × 0.32

Data collection	
Diffractometer	Bruker D8 QUEST PHOTON 3 diffractometer
Absorption correction	Numerical (*SADABS*; Krause *et al.*, 2015 ▸)
*T*_min_, *T*_max_	0.93, 0.97
No. of measured, independent and observed [*I* > 2σ(*I*)] reflections	80207, 6020, 5304
*R*_int_	0.025
(sin θ/λ)_max_ (Å^−1^)	0.774

Refinement	
*R*[*F* ^2^ > 2σ(*F* ^2^)], *wR*(*F* ^2^), *S*	0.046, 0.133, 1.03
No. of reflections	6020
No. of parameters	217
H-atom treatment	H-atom parameters constrained
Δρ_max_, Δρ_min_ (e Å^−3^)	0.46, −0.21

Computer programs: *APEX3* and *SAINT* (Bruker, 2020 ▸), *SHELXT* (Sheldrick, 2015*a* ▸), *SHELXL2018/1* (Sheldrick, 2015*b* ▸), *DIAMOND* (Brandenburg & Putz, 2012 ▸) and *SHELXTL* (Sheldrick, 2008 ▸).

## supplementary crystallographic information

### Crystal data

**Table:**

C_20_H_15_NO_3_	*F*(000) = 664
*M**_r_* = 317.33	*D*_x_ = 1.344 Mg m^−^^3^
Monoclinic, *P*2_1_/*n*	Mo *K*α radiation, λ = 0.71073 Å
*a* = 8.2284 (3) Å	Cell parameters from 9939 reflections
*b* = 13.7693 (4) Å	θ = 2.5–33.3°
*c* = 13.9230 (4) Å	µ = 0.09 mm^−^^1^
β = 96.155 (1)°	*T* = 150 K
*V* = 1568.37 (9) Å^3^	Block, colourless
*Z* = 4	0.44 × 0.35 × 0.32 mm

### Data collection

**Table:**

Bruker D8 QUEST PHOTON 3 diffractometer	6020 independent reflections
Radiation source: fine-focus sealed tube	5304 reflections with *I* > 2σ(*I*)
Graphite monochromator	*R*_int_ = 0.025
Detector resolution: 7.3910 pixels mm^-1^	θ_max_ = 33.4°, θ_min_ = 2.9°
φ and ω scans	*h* = −12→12
Absorption correction: numerical (*SADABS*; Krause *et al.*, 2015)	*k* = −21→21
*T*_min_ = 0.93, *T*_max_ = 0.97	*l* = −21→21
80207 measured reflections	

### Refinement

**Table:**

Refinement on *F*^2^	Primary atom site location: dual
Least-squares matrix: full	Secondary atom site location: difference Fourier map
*R*[*F*^2^ > 2σ(*F*^2^)] = 0.046	Hydrogen site location: inferred from neighbouring sites
*wR*(*F*^2^) = 0.133	H-atom parameters constrained
*S* = 1.03	*w* = 1/[σ^2^(*F*_o_^2^) + (0.0739*P*)^2^ + 0.3685*P*] where *P* = (*F*_o_^2^ + 2*F*_c_^2^)/3
6020 reflections	(Δ/σ)_max_ = 0.001
217 parameters	Δρ_max_ = 0.46 e Å^−^^3^
0 restraints	Δρ_min_ = −0.21 e Å^−^^3^

### Special details

**Table:**

Experimental. The diffraction data were obtained from 9 sets of frames, each of width 0.5° in ω or φ, collected with scan parameters determined by the "strategy" routine in *APEX3*. The scan time was 7 sec/frame.
Geometry. All esds (except the esd in the dihedral angle between two l.s. planes) are estimated using the full covariance matrix. The cell esds are taken into account individually in the estimation of esds in distances, angles and torsion angles; correlations between esds in cell parameters are only used when they are defined by crystal symmetry. An approximate (isotropic) treatment of cell esds is used for estimating esds involving l.s. planes.
Refinement. Refinement of F^2^ against ALL reflections. The weighted R-factor wR and goodness of fit S are based on F^2^, conventional R-factors R are based on F, with F set to zero for negative F^2^. The threshold expression of F^2^ > 2sigma(F^2^) is used only for calculating R-factors(gt) etc. and is not relevant to the choice of reflections for refinement. R-factors based on F^2^ are statistically about twice as large as those based on F, and R- factors based on ALL data will be even larger. H-atoms attached to carbon were placed in calculated positions (C—H = 0.95 - 1.00 Å). All were included as riding contributions with isotropic displacement parameters 1.2 - 1.5 times those of the attached atoms. Two reflections affected by the beamstop were omitted from the final refinement.

### Fractional atomic coordinates and isotropic or equivalent isotropic displacement parameters (Å^2^)

**Table:**

	*x*	*y*	*z*	*U*_iso_*/*U*_eq_	
O1	0.79130 (8)	0.56932 (5)	0.29079 (6)	0.03386 (17)	
O2	0.15346 (8)	0.39853 (5)	0.36012 (6)	0.03159 (16)	
O3	0.34027 (8)	0.35057 (5)	0.26450 (5)	0.02547 (14)	
N1	0.63622 (8)	0.63640 (5)	0.39987 (5)	0.01811 (12)	
C1	0.49088 (9)	0.64053 (5)	0.44357 (5)	0.01656 (13)	
C2	0.46483 (10)	0.71713 (6)	0.50694 (6)	0.02098 (14)	
H2	0.544815	0.766658	0.518906	0.025*	
C3	0.32280 (11)	0.72050 (6)	0.55192 (6)	0.02469 (16)	
H3	0.306866	0.771800	0.595567	0.030*	
C4	0.20287 (10)	0.64925 (7)	0.53375 (6)	0.02508 (16)	
H4	0.106080	0.651845	0.565229	0.030*	
C5	0.22538 (10)	0.57466 (6)	0.46961 (6)	0.02108 (14)	
H5	0.142234	0.527154	0.456509	0.025*	
C6	0.36952 (9)	0.56798 (5)	0.42340 (5)	0.01629 (13)	
C7	0.40167 (8)	0.49162 (5)	0.35579 (5)	0.01659 (13)	
C8	0.54184 (9)	0.49270 (6)	0.31341 (6)	0.02043 (14)	
H8	0.560136	0.442273	0.269253	0.025*	
C9	0.66584 (10)	0.56733 (6)	0.33219 (6)	0.02148 (15)	
C10	0.76472 (10)	0.71018 (6)	0.42091 (6)	0.02241 (15)	
H10A	0.775440	0.725750	0.490720	0.027*	
H10B	0.870536	0.683646	0.405222	0.027*	
C11	0.72727 (11)	0.79935 (6)	0.36498 (7)	0.02598 (17)	
C12	0.69298 (13)	0.87027 (8)	0.31880 (9)	0.0356 (2)	
H12	0.665529	0.927061	0.281827	0.043*	
C13	0.28317 (9)	0.41024 (5)	0.32886 (6)	0.01886 (14)	
C14	0.23208 (11)	0.27253 (7)	0.22635 (6)	0.02652 (17)	
H14A	0.266692	0.250123	0.164117	0.032*	
H14B	0.119369	0.298095	0.213628	0.032*	
C15	0.23223 (10)	0.18782 (6)	0.29404 (6)	0.02316 (15)	
C16	0.36596 (12)	0.12502 (8)	0.30625 (7)	0.0318 (2)	
H16	0.457268	0.135594	0.271197	0.038*	
C17	0.36579 (17)	0.04679 (9)	0.36979 (9)	0.0430 (3)	
H17	0.457907	0.004905	0.378888	0.052*	
C18	0.2320 (2)	0.02998 (9)	0.41959 (9)	0.0502 (3)	
H18	0.232480	−0.023368	0.462895	0.060*	
C19	0.09713 (18)	0.09064 (9)	0.40660 (9)	0.0442 (3)	
H19	0.004674	0.078535	0.440243	0.053*	
C20	0.09774 (12)	0.16940 (7)	0.34402 (7)	0.02989 (19)	
H20	0.005332	0.211073	0.335298	0.036*	

### Atomic displacement parameters (Å^2^)

**Table:**

	*U*^11^	*U*^22^	*U*^33^	*U*^12^	*U*^13^	*U*^23^
O1	0.0257 (3)	0.0298 (3)	0.0496 (4)	−0.0088 (3)	0.0203 (3)	−0.0130 (3)
O2	0.0222 (3)	0.0270 (3)	0.0474 (4)	−0.0083 (2)	0.0127 (3)	−0.0108 (3)
O3	0.0263 (3)	0.0234 (3)	0.0276 (3)	−0.0089 (2)	0.0067 (2)	−0.0090 (2)
N1	0.0171 (3)	0.0150 (3)	0.0224 (3)	−0.0026 (2)	0.0026 (2)	−0.0004 (2)
C1	0.0176 (3)	0.0148 (3)	0.0171 (3)	0.0000 (2)	0.0009 (2)	0.0012 (2)
C2	0.0242 (3)	0.0177 (3)	0.0208 (3)	−0.0006 (3)	0.0015 (3)	−0.0023 (2)
C3	0.0270 (4)	0.0233 (4)	0.0240 (3)	0.0028 (3)	0.0041 (3)	−0.0051 (3)
C4	0.0219 (3)	0.0277 (4)	0.0265 (4)	0.0025 (3)	0.0070 (3)	−0.0035 (3)
C5	0.0182 (3)	0.0223 (3)	0.0232 (3)	0.0000 (2)	0.0040 (2)	−0.0010 (3)
C6	0.0160 (3)	0.0154 (3)	0.0174 (3)	0.0005 (2)	0.0011 (2)	0.0012 (2)
C7	0.0165 (3)	0.0145 (3)	0.0186 (3)	−0.0011 (2)	0.0013 (2)	0.0007 (2)
C8	0.0196 (3)	0.0164 (3)	0.0260 (3)	−0.0028 (2)	0.0059 (3)	−0.0032 (2)
C9	0.0191 (3)	0.0178 (3)	0.0284 (4)	−0.0025 (2)	0.0068 (3)	−0.0027 (3)
C10	0.0197 (3)	0.0192 (3)	0.0281 (4)	−0.0051 (3)	0.0013 (3)	−0.0009 (3)
C11	0.0237 (3)	0.0219 (4)	0.0332 (4)	−0.0062 (3)	0.0072 (3)	0.0002 (3)
C12	0.0313 (4)	0.0291 (4)	0.0484 (6)	−0.0021 (4)	0.0138 (4)	0.0106 (4)
C13	0.0183 (3)	0.0167 (3)	0.0213 (3)	−0.0018 (2)	0.0008 (2)	−0.0001 (2)
C14	0.0295 (4)	0.0249 (4)	0.0247 (4)	−0.0092 (3)	0.0008 (3)	−0.0067 (3)
C15	0.0230 (3)	0.0217 (3)	0.0250 (3)	−0.0049 (3)	0.0034 (3)	−0.0082 (3)
C16	0.0268 (4)	0.0337 (5)	0.0345 (4)	0.0020 (3)	0.0016 (3)	−0.0126 (4)
C17	0.0522 (7)	0.0316 (5)	0.0426 (6)	0.0117 (5)	−0.0067 (5)	−0.0085 (4)
C18	0.0824 (10)	0.0299 (5)	0.0385 (6)	−0.0013 (6)	0.0066 (6)	0.0026 (4)
C19	0.0619 (7)	0.0333 (5)	0.0409 (6)	−0.0112 (5)	0.0214 (5)	−0.0035 (4)
C20	0.0309 (4)	0.0253 (4)	0.0355 (4)	−0.0054 (3)	0.0123 (3)	−0.0084 (3)

### Geometric parameters (Å, º)

**Table:**

O1—C9	1.2355 (10)	C8—H8	0.9500
O2—C13	1.2058 (10)	C10—C11	1.4687 (12)
O3—C13	1.3375 (10)	C10—H10A	0.9900
O3—C14	1.4588 (10)	C10—H10B	0.9900
N1—C9	1.3788 (10)	C11—C12	1.1865 (14)
N1—C1	1.3999 (10)	C12—H12	0.9500
N1—C10	1.4730 (10)	C14—C15	1.4995 (13)
C1—C2	1.4062 (10)	C14—H14A	0.9900
C1—C6	1.4192 (10)	C14—H14B	0.9900
C2—C3	1.3846 (11)	C15—C20	1.3922 (12)
C2—H2	0.9500	C15—C16	1.3955 (13)
C3—C4	1.3953 (12)	C16—C17	1.3940 (17)
C3—H3	0.9500	C16—H16	0.9500
C4—C5	1.3864 (11)	C17—C18	1.382 (2)
C4—H4	0.9500	C17—H17	0.9500
C5—C6	1.4116 (10)	C18—C19	1.385 (2)
C5—H5	0.9500	C18—H18	0.9500
C6—C7	1.4543 (10)	C19—C20	1.3914 (16)
C7—C8	1.3507 (10)	C19—H19	0.9500
C7—C13	1.5062 (10)	C20—H20	0.9500
C8—C9	1.4520 (11)		
			
C13—O3—C14	116.87 (7)	N1—C10—H10A	109.3
C9—N1—C1	123.16 (6)	C11—C10—H10B	109.3
C9—N1—C10	115.85 (6)	N1—C10—H10B	109.3
C1—N1—C10	120.93 (6)	H10A—C10—H10B	108.0
N1—C1—C2	119.87 (7)	C12—C11—C10	178.18 (10)
N1—C1—C6	120.08 (6)	C11—C12—H12	180.0
C2—C1—C6	120.05 (7)	O2—C13—O3	123.21 (7)
C3—C2—C1	120.12 (7)	O2—C13—C7	125.74 (7)
C3—C2—H2	119.9	O3—C13—C7	111.05 (6)
C1—C2—H2	119.9	O3—C14—C15	112.63 (7)
C2—C3—C4	120.62 (7)	O3—C14—H14A	109.1
C2—C3—H3	119.7	C15—C14—H14A	109.1
C4—C3—H3	119.7	O3—C14—H14B	109.1
C5—C4—C3	119.80 (8)	C15—C14—H14B	109.1
C5—C4—H4	120.1	H14A—C14—H14B	107.8
C3—C4—H4	120.1	C20—C15—C16	119.00 (9)
C4—C5—C6	121.24 (7)	C20—C15—C14	120.58 (8)
C4—C5—H5	119.4	C16—C15—C14	120.41 (8)
C6—C5—H5	119.4	C17—C16—C15	120.11 (10)
C5—C6—C1	118.15 (7)	C17—C16—H16	119.9
C5—C6—C7	124.21 (7)	C15—C16—H16	119.9
C1—C6—C7	117.65 (6)	C18—C17—C16	120.18 (11)
C8—C7—C6	119.87 (6)	C18—C17—H17	119.9
C8—C7—C13	117.37 (7)	C16—C17—H17	119.9
C6—C7—C13	122.76 (6)	C17—C18—C19	120.23 (11)
C7—C8—C9	123.06 (7)	C17—C18—H18	119.9
C7—C8—H8	118.5	C19—C18—H18	119.9
C9—C8—H8	118.5	C18—C19—C20	119.68 (11)
O1—C9—N1	121.42 (7)	C18—C19—H19	120.2
O1—C9—C8	122.54 (7)	C20—C19—H19	120.2
N1—C9—C8	116.04 (7)	C19—C20—C15	120.76 (10)
C11—C10—N1	111.46 (7)	C19—C20—H20	119.6
C11—C10—H10A	109.3	C15—C20—H20	119.6
			
C9—N1—C1—C2	−175.56 (7)	C1—N1—C9—C8	−4.77 (11)
C10—N1—C1—C2	1.37 (10)	C10—N1—C9—C8	178.16 (7)
C9—N1—C1—C6	3.97 (11)	C7—C8—C9—O1	−177.70 (9)
C10—N1—C1—C6	−179.10 (7)	C7—C8—C9—N1	2.64 (12)
N1—C1—C2—C3	−178.65 (7)	C9—N1—C10—C11	96.66 (8)
C6—C1—C2—C3	1.82 (11)	C1—N1—C10—C11	−80.48 (9)
C1—C2—C3—C4	−1.16 (13)	C14—O3—C13—O2	4.71 (12)
C2—C3—C4—C5	−0.41 (13)	C14—O3—C13—C7	−175.41 (7)
C3—C4—C5—C6	1.33 (13)	C8—C7—C13—O2	179.78 (8)
C4—C5—C6—C1	−0.66 (11)	C6—C7—C13—O2	−1.07 (12)
C4—C5—C6—C7	179.70 (7)	C8—C7—C13—O3	−0.10 (10)
N1—C1—C6—C5	179.56 (7)	C6—C7—C13—O3	179.05 (7)
C2—C1—C6—C5	−0.91 (11)	C13—O3—C14—C15	−80.18 (10)
N1—C1—C6—C7	−0.77 (10)	O3—C14—C15—C20	107.82 (9)
C2—C1—C6—C7	178.76 (7)	O3—C14—C15—C16	−73.57 (10)
C5—C6—C7—C8	178.41 (7)	C20—C15—C16—C17	−1.82 (13)
C1—C6—C7—C8	−1.23 (11)	C14—C15—C16—C17	179.56 (8)
C5—C6—C7—C13	−0.72 (11)	C15—C16—C17—C18	1.23 (16)
C1—C6—C7—C13	179.64 (6)	C16—C17—C18—C19	0.10 (18)
C6—C7—C8—C9	0.28 (12)	C17—C18—C19—C20	−0.80 (19)
C13—C7—C8—C9	179.45 (7)	C18—C19—C20—C15	0.19 (17)
C1—N1—C9—O1	175.57 (8)	C16—C15—C20—C19	1.12 (14)
C10—N1—C9—O1	−1.50 (12)	C14—C15—C20—C19	179.74 (9)

### Hydrogen-bond geometry (Å, º)

*Cg*2 and *Cg*3 are the centroids of the C1–C6 and C15–C20 benzene rings, respectively.

**Table:**

*D*—H···*A*	*D*—H	H···*A*	*D*···*A*	*D*—H···*A*
C2—H2···*Cg*3^i^	0.95	2.94	3.8206 (10)	154
C4—H4···O2^ii^	0.95	2.57	3.4846 (11)	162
C5—H5···O2	0.95	2.23	2.8917 (11)	126
C12—H12···O1^iii^	0.95	2.25	3.1463 (14)	157
C14—H14*A*···*Cg*2^iv^	0.99	2.65	3.4652 (9)	140
C16—H16···O1^v^	0.95	2.50	3.3443 (12)	148

Symmetry codes: (i) −*x*+1, −*y*+1, −*z*+1; (ii) −*x*, −*y*+1, −*z*+1; (iii) −*x*+3/2, *y*+1/2, −*z*+1/2; (iv) −*x*+1/2, *y*−1/2, −*z*+1/2; (v) −*x*+3/2, *y*−1/2, −*z*+1/2.
